# 2-Heteroarylethylamines in medicinal chemistry: a review of 2-phenethylamine satellite chemical space

**DOI:** 10.3762/bjoc.20.163

**Published:** 2024-08-02

**Authors:** Carlos Nieto, Alejandro Manchado, Ángel García-González, David Díez, Narciso M Garrido

**Affiliations:** 1 Department of Organic Chemistry, Faculty of Chemical Sciences, University of Salamanca, Pl. Caídos, s/n, 37008 Salamanca, Spain

**Keywords:** bioisosteres, 2-heteroarylethylamines, medicinal chemistry, 2-phenethylamine, scaffold hopping

## Abstract

The concept of bioisostere replacement is of paramount importance in medicinal chemistry, as it can be employed as a rational to expand bioactive chemical space to tackle lead optimization issues like lack of potency, efficacy, and selectivity or pharmacokinetic/dynamic issues. One of the most important building blocks (in the sense of participating in a vast area of chemical space of biological importance) in medicinal chemistry is the 2-phenethyl moiety, a key component of diverse drug-like entities. Although the core 2-phenethylamine structure has been recognized by the drug discovery community, little attention has been given to the various ring-based rescaffolding procedures that can be conducted with this unit. In this regard, a review on the use of 2-heteroarylethylamines displaying pharmacological activity is reported. A detailed description of flexible, amine-opened motifs is provided, that describes therapeutic targets and other potent bioactive examples, which will be a valuable repository of phenyl, heteroaryl, and other replacement units of high value to the drug discovery community.

## Introduction

One of the major hit-2-lead exploration techniques in any medicinal chemistry program – knowledge-based or computationally aided – is bioisosteric replacement, where a particular arrangement of atoms, such as a functional group, chain, ring, linker, etc., is substituted by motifs with size, electronic, and physicochemical characteristics comparable to the original [1]. The main purpose of this approach is quality improvement, such as activity, selectivity, bioavailability, metabolism, and/or toxicity, while expanding the chemical space surrounding bioactive compounds [2–3]. Benzene-to-heteroaromatic ring replacement represents a classical structural hopping strategy, as five- or six-membered heterocyclic aromatic rings are widespread entities in drug discovery [4]. In this sense, any bioactive molecule enclosing a benzene ring in its initial optimization stages could undergo a heteroaromatic replacement.

**Scheme 1:**
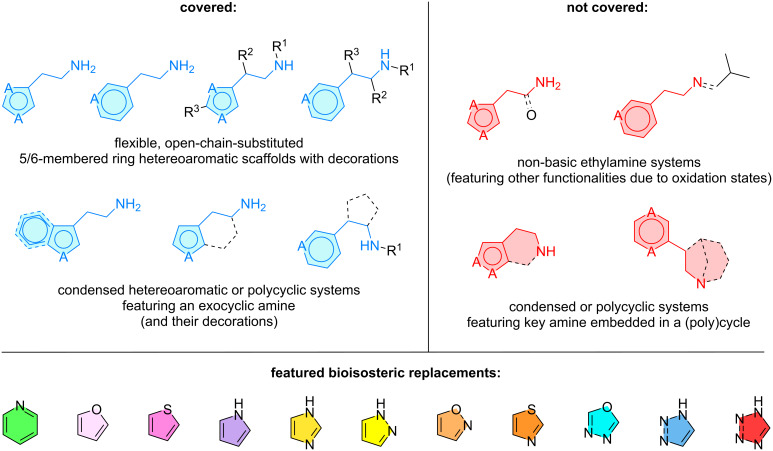
Description of the 2-heteroarylethylamine scope of the present review featuring appropriate heteroaromatic systems (A = O, N, S).

2-Phenethylamines are notable bioactive compounds towards different disease-related receptors, as it was described in our previous work [5]. By means of benzene ring aromatic rescaffolding, it is possible to access the 2‑heteroarylethylamine neighboring space. This satellite chemical region is rich not only in structures displaying affinity to key phenethylamine targets like adrenergic or histamine-type receptors, but also to novel ones such as TAAR1 (trace-amine-associated receptor 1), σ1/2 (sigma receptors 1 and 2), or AChE (acetylcholinesterase). Similar to our previous review, a descriptive, simple scope is presented below to outline which structural motifs are included in this work and which ones are discarded (Scheme 1). In detail, this review encompasses bioactive compounds which satisfy:

- flexible, open chain-substituted 5/6-membered heteroaromatic scaffolds with decorations,
- condensed heteroaromatic or polycyclic systems featuring an exocyclic amine (and their substitutions).

Systems out of scope of this review are those, where:

- non-basic ethylamine systems are present (featuring other functionalities due to oxidation states),
- condensed or polycyclic systems are present and featuring a key amine embedded in a (poly)cycle.

Consequently, a dedicated review covering the 2‑heteroarylethylamine space is presented here. This work is divided into subsections covering individual heteroaryl replacements and target bioactive deployment, rather than a pure disease-related target division as in our previous review. The absence of a specific heteroaromatic subsection indicates no biologically relevant data has been reported up to date.

## Review

### 2‑Heteroarylethylamine scaffolds of biological importance

#### Six-membered heteroaromatic rings

**Scheme 2:**
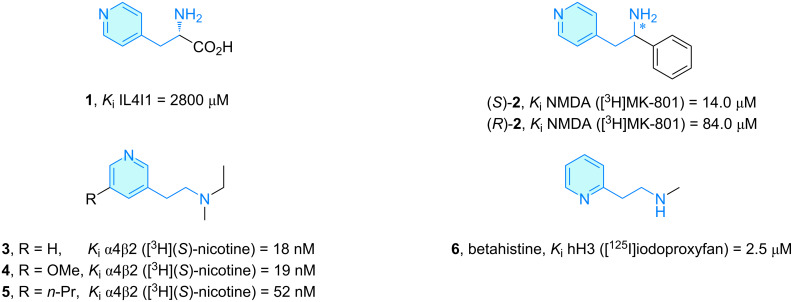
2-Aminoethylpyridine derivatives with therapeutic activity.

**Pyridines: ***IL4I1*, interleukin-4 induced gene 1, encodes ʟ-phenylalanine oxidase IL4I1 present in the tumor bed of a vast diversity of human tumor types. As phenylalanine is the preferred substrate of IL4I1 catalytic activity, Presset et al. [6] reported novel phenylalanine derivatives as a strategy to inhibit IL4I1 activity, as this enzyme has a preference for hydrophobic amino acids. Among them, commercial compound **1** (Scheme 2) represents a rescaffolding exercise to pyridine retaining low inhibitory activity although it was found toxic in in vitro assays on a human T-cell line and PBMCs (periferal blood mononuclear cells).

The derivatives (*R*)-**2** and (*S*)-**2** were elaborated by Berger et al. [7] in the course of an extensive screening of NMDA (*N*-methyl-ᴅ-aspartate) channel blockers resembling 1,2-diphenylethylamines. A channel pharmacophore description was envisaged collecting data from stereoisomers of 1,2-diphenylethylamine derivatives and 1,2-dicyclohexylethylamine derivatives. Among them, isomer (*S*)-**2**, also called lanicemine, AZD6765 or AR-R15896AR, was described as a competitive ketamine alternative without psychotomimetic side effects, although potency and selectivity were significantly lower (Scheme 2) [8–9].

Dukat et al. [10] developed flexible 3-(2-aminoethyl)pyridine (AEP) analogs **3**–**5** as α4β2 nicotinic cholinergic receptor ligands with nanomolar activities in rat brain homogenates (Scheme 2). The idea behind these AEP structures was to check activity correlation against a nicotine series. The comparison of *K*_i_ values of both series showed a moderate correlation, which opens the possibility of different binding topologies to the α4β2 receptor.

Betahistine (**6**) is an orally active 2-(2-aminoethyl)pyridine drug indicated for vestibular disorders like Meniere’s disease, whose patients exhibit acute vertigo attacks (Scheme 2) [11–12]. Gbahou et al. [13] demonstrated histaminergic synapse improvement through inverse agonism at histamine receptor 3 (H_3_) using recombinant isoforms. This finding corrects their previous assumption of betahistidine acting as an antagonist. Inhibition of cAMP formation and [^3^H]arachidonic acid release concluded the inverse agonist role.

#### Five-membered heteroaromatic rings

**Scheme 3:**
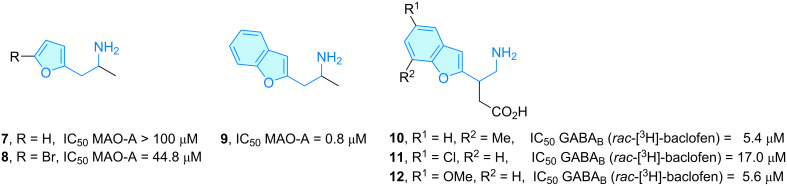
2-Aminoethylfuran derivatives with therapeutic activity.

**Furans:** Racemic heteroarylisopropylamines were described as MAO inhibitors (monoamine oxidase) by Vallejos et al. [14] as a natural extension of their previous QSAR studies using phenylisopropylamines [15]. The authors supported their aryl-to-heteroaryl group hopping due to the success of similar replacements leading to novel MAO-A bioactive entities [16–17]. The brominated analogue **8** showed moderate MAO-A activity compared to the parent 2-furyl compound **7**, as a result of increased polarizability. The condensed benzofuran **9** revealed submicromolar MAO-A potency, a resemblance to the indole system of 5-hydroxytryptamine (a MAO-A substrate), in a molecular docking experiment tested against serotonin (Scheme 3).

A similar class of benzofuran systems with attractive binding properties are those represented by 4-amino-3-(benzo[*b*]furan-2-yl)butanoic acids, baclofen analogs, elaborated to elucidate the structural requirements for access to γ-aminobutyric receptor type B (GABA_B_) [18]. Amino acids **10**–**12** were demonstrated [19–21] to act as substrates of GABA_B_ (Scheme 3), key metabotropic receptors from the G-protein-coupled receptor superfamily responsible for CNS inhibitory synapses [22]. The authors concluded that a heteroaromatic ring bound to the C3 position of the GABA chain is well tolerated for activity.

**Scheme 4:**
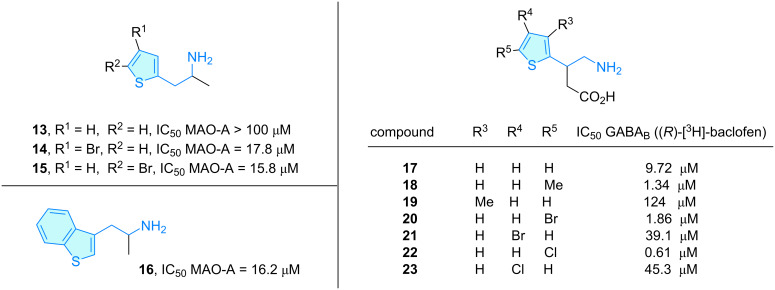
2-Aminoethylthiophene derivatives with therapeutic activity, part 1.

**Thiophenes:** Back to the MAO-A scenario, Vallejo et al. [14] also developed thienyl-substituted isopropylamines **13**–**16**, which were found to bind to MAO-A with IC_50_ values in the micromolar range with better inhibitory data than for the aforementioned furyl analogues (Scheme 4). The authors suggested the replacement of furyl by a more polarizable aromatic ring such as thienyl as prospective origin of the observed IC_50_ downward shift.

Berthelot et al. [23–24] expanded their studies on GABA_B_ inhibitors from furanyl derivatives to thienyl-substituted compounds **17**–**23** in the search to elucidate structural features for accessing this receptor (Scheme 4). The chloride- and bromide-substituted thienyl derivatives encompassed micromolar to submicromolar activities in radioligand binding assays based on (*R*)-[^3^H]-baclofen displacement. QSAR studies have been developed in order to examine the pivotal role of the aromatic moiety of baclofen-like compounds [25]. In this sense, the QSAR equation revealed HOMO/LUMO orbital energies are critical for a high correlation with binding strength.

**Scheme 5:**
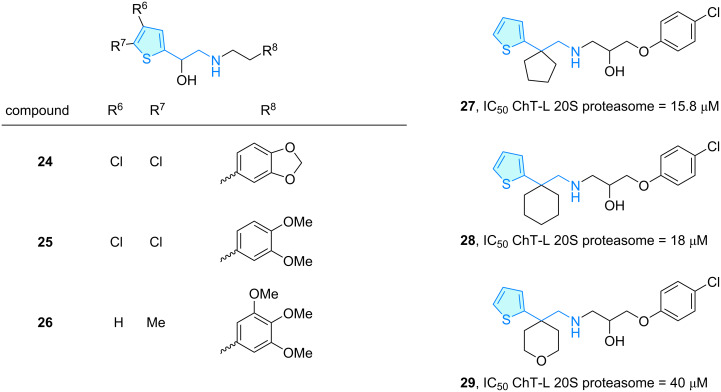
2-Aminoethylthiophene derivatives with therapeutic activity, part 2.

An in vivo antihypertensive activity was demonstrated for a series of flexible secondary amines incorporating terminal aromatic rings by Bagli et al. [26]. A blood pressure lowering effect was observed for 2-hydroxy-2-thienylethylamines **24**–**26** (Scheme 5), which was related to typical antihypertensive pathways like adrenergic system interference, catecholamine depletion, or plasma volume lowering.

The 20S proteasome core particle represents a critical degradation machinery for cellular homeostasis [27]. A set of (thiophen-2-yl)cycloalkyl(phenoxypropanol)amines **27**–**29** was tested against caspase-like and chymotrypsin-like activities of this supramolecular complex (Scheme 5), with positive in vitro activities in 20S proteasome core particles isolated from rabbit erythrocytes [28].

**Scheme 6:**
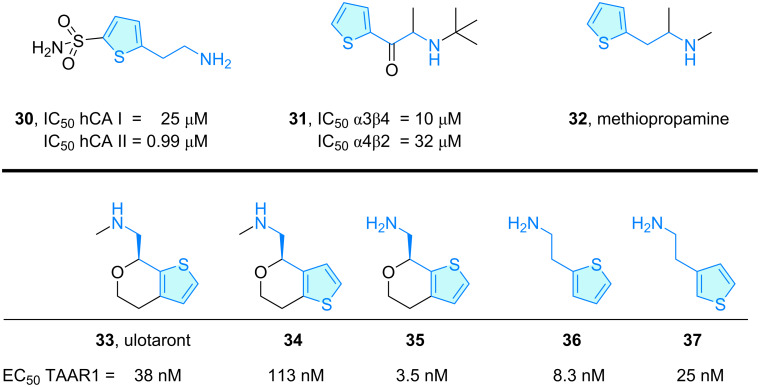
2-Aminoethylthiophene derivatives with therapeutic activity, part 3.

The sulfonamide **30** (Scheme 6) has been evaluated as inhibitor of human carbonic anhydrase I/II (hCA I and II), which catalyze the reversible hydration reaction of carbon dioxide to bicarbonate, cyanates to carbamic acids, aldehydes to *gem*-diols, etc., and represent a potential therapeutic target for diseases like osteoporosis, edema, obesity or cancer [29]. Alım et al. [30] evaluated a series of thiophene sulfonamides based on the high stability of this aromatic ring. Molecular docking studies combined with in vitro studies showed that only the thiophene-based phenethylamine derivative **30** possesses a weak hCA I/II activity compared with analogues lacking the 2-aminoethyl moiety.

Carrol et al. [31–32] explored bupropion analogues for their capacity to antagonize human nAChRs (nicotinic acetylcholine receptor) as these are of clinical importance in developing tobacco-derived diseases. From the range of propiophenone derivatives elaborated tackling dopaminergic activities, thiophene **31** showed weak inhibitory activity towards α3β4 and α4β2 (Scheme 6).

Methiopropamine (**32**) [33] is an emergent psychoactive substance structurally similar to methamphetamine, where the aromatic moiety was rescaffolded from benzene to thiophene (Scheme 6). First synthesized in 1942 [34], it irrupted into the recreational drug market in 2011 [35], with acute toxicity reports in hospital admissions. Nguyen et al. [36] investigated its effects in mice, demonstrating neurotoxicity via dopamine receptors, while Tuv et al. [37] studied the compound’s phamarcokinetics, pharmacodynamics, and mode of action in comparison to methamphetamine, which revealed a significantly lower potency of **32**.

Ulotaront (**33**, SEP-363856) [38] is a phase-3 clinical lead for the treatment of schizophrenia, displaying TAAR1 (trace-amine-associated receptor 1) [39] and 5-HT1A agonism as mode of action, lacking dopamine D2 and 5-HT2A antagonism. SAR exploration of the ulotaront family was envisaged by Heffernan et al. [40], including human TAAR1 agonist activity and structural evaluation via homology model development followed by molecular docking and molecular dynamics studies (Scheme 6). Structural features like sulfur location and ring opening of the aminoethyl section were investigated computationally, identifying key interactions to understand TAAR1 agonism.

**Scheme 7:**
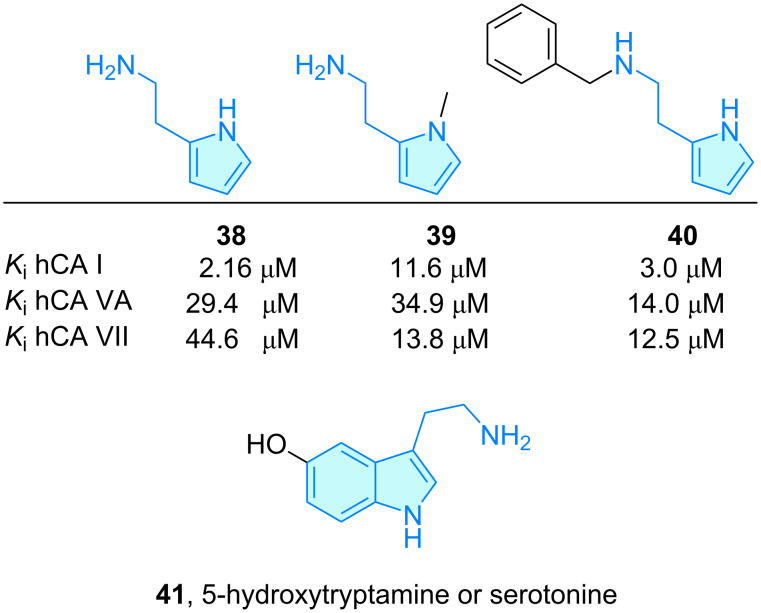
2-Aminoethylpyrrole derivatives with therapeutic activity.

**Pyrroles:** New histamine-related compounds were synthesized and evaluated towards activation of human carbonic anhydrase isoforms (hCA), aiming at potency and selectivity enhancement by Chiaramonte et al. [41]. Among them, a discrete set of 2-aminoethylpyrrole (Scheme 7) hits were elaborated and tested in a stopped-flow CO_2_ hydrase assay. Comparing this pyrrole family with original histamine inferred a decrease in selectivity towards the hCA VII isoform, while activity was not affected significantly.

Tryptamine derivatives, i.e., compounds derived from 2-(indole-3-yl)ethylamine, comprise a huge number of indole compounds such as serotonin (**41**) [42] (Scheme 7). These compounds play an important role for a variety of biological targets, from 5-HT (serotonin receptors) to RAS [43–44], and are used to treat disorders as diverse as obesity, oncology, CNS disorders, etc. Following these overwhelming features, these are not included in this work as capturing an adequate group of representatives, even selecting only the most prominent ones, would mask other heteroaromatic structures.

**Scheme 8:**
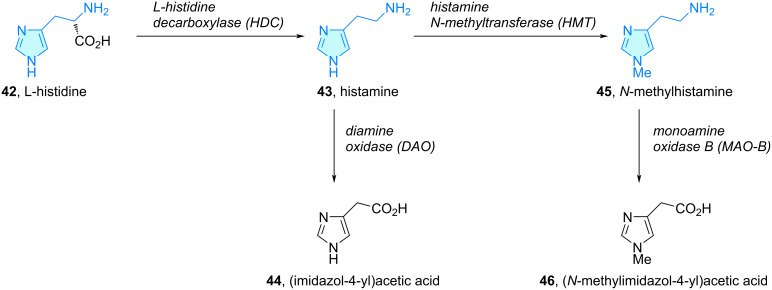
Histamine metabolic pathway.

**Imidazoles:** Probably, the pinnacle of the 2-heteroarylethylamine chemical space is constituted by the biogenic amine histamine (**43**). In a similar fashion as dopamine and epinephrine produced from ʟ-phenylalanine along the catecholamine pathway, histamine is generated from the amino acid ʟ-histidine (**42**) via enzymatic decarboxylation promoted by ʟ-histidine decarboxylase (Scheme 8) [45–47]. Histamine is commonly degradated by two enzymes: diamine oxidase (DAO) to produce (imidazol-4-yl)acetic acid (**44**), or histamine *N*-methyltransferase (HMT) to *N*-methylhistamine **45**. Monoamine oxidase B (MAO-B) transforms *N*-methylhistamine into (*N*-methylimidazol-4-yl)acetic acid (**46**). The major source of histamine are mast cells, although it is additionally biosynthesized in basophils, other immune cells, and tissues like intestinal mucosa, skin, or the heart [45,48].

**Scheme 9:**
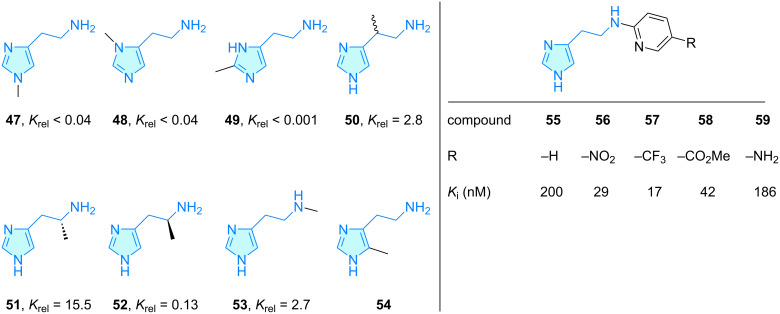
2-Aminoethylimidazole derivatives with therapeutic activity, part 1. *K*_rel_ is referred as histamine relative potency (basal reference 1.0).

Histamine plays many pivotal roles in the onset of allergies via Th2 cytokine secretion and inhibition of Th1 cytokine, leukotriene and chemokines release, or IL-6 induction. Histamine targets histamine receptors H1–4, triggering pro-inflammatory or anti-inflammatory events depending on the receptor type and cells involved [45,49]. Histamine also plays a critical role in both vertebrates and invertebrates as neurotransmitter, in the so called histaminergic synapses [50]. As a consequence, histamine has been used as a template to rationally design histamine receptor agonists/antagonists capable to modulate their extensive range of capabilities.

(*R*)-α-Methylhistamine (**51**) is an H_3_ receptor agonist approximately 15-fold more active than histamine (Scheme 9). Gannellin et al. [51] performed a discrete H_3_ SAR study starting from compound **51** and investigated the effect of the position of the methyl group on the agonist activity. Analogues **50** and **53**, having the methyl group in the aminoethyl side chain, showed almost a 3-fold potency compared to histamine, while the derivatives **47**–**49** with a methyl group attached to the imidazole core demonstrated lower relative potency. The derivative **54** was investigated towards its effect against the H_4_ receptor, recently [52–54]. Furthermore, an antagonistic H_3_ SAR study was achieved creating *N*-arylimidazolethylamine counterparts **55**–**59** (Scheme 9). It was shown, that electron-withdrawing substituents at the pyridine 5-position lowered the antagonistic activity for this small family.

**Scheme 10:**
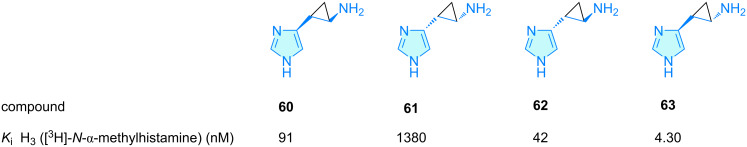
Conformationally restricted 2-aminoethylimidazole derivatives with therapeutic activity, part 2.

Conformationally restricted cyclopropylhistamine analogues were disclosed by De Esch et al. [55] and by Kazuta el al. [56]. The primary aim was the design of a new class of highly H_3_-selective agonists lacking H_4_ affinity by restricting the flexibility of the aminoethyl chain. Previous structure–activity exercises demonstrated an impact on selectivity upon introducing a stereocenter into the flexible aminoethyl chain [57]. Radioligand binding assays showed activities in the nanomolar range for the four diastereomers, while functional assays demonstrated only H_3_-subtype activity for compound **63**, with no H_4_ subtype activity, which was the original goal (Scheme 10).

**Scheme 11:**
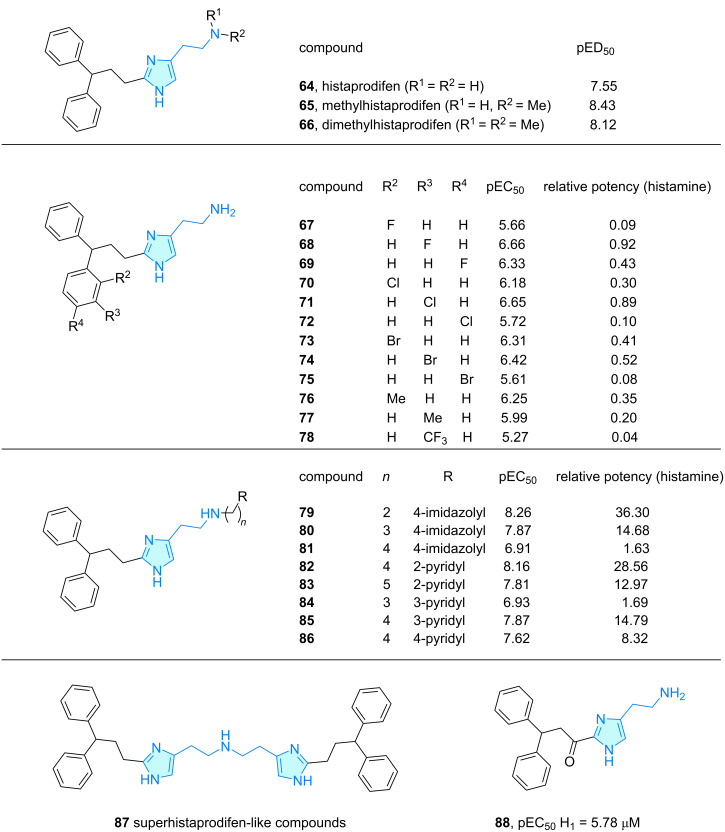
2-Aminoethylimidazole derivatives with therapeutic activity, part 3.

Histaprodifen **64** is a potent H_1_ receptor agonist with a 3,3-diphenylpropyl moiety at position 2 of the imidazole ring characterized by Elz and co-workers [58]. The authors showed parent compound **64** and methylated derivatives **65** and **66** were potent H_1_ receptor agonists in pithed and anaesthetized rats (Scheme 11). Later, the authors expanded the histaprodifen family by SAR exploration of small substituents in the phenyl rings (compounds **67**–**78**, Scheme 11) [59–60]. While pEC_50_ values varied very subtle, a histamine relative potency screening revealed a general reduction in potency. Following the same assay, Menghin and co-workers [61] explored flexible chain incorporations at the terminal nitrogen of histaprodifen generating hits **79**–**86** (Scheme 11). Finally, the *N*,*N*-bis(2-imidazolyl)ethyl)-substituted amine superhistaprodifen **87** cluster was exhaustively synthesized and tested for their agonist activity against the H_1_ receptor by Straßer et al. [62]. From the set of compounds, biological assays revealed p*K*_i_ values of 4.5–7.5 in human, rat, bovine, and guinea pig H_1_ receptor activities. Additional modelling studies via CoMFA (comparative molecular field analysis) and posterior comparison with experimental data showed good agreement, suggesting two different binding topologies. Patil et al. [63] synthesized compound **88**, an oxidized version at position 1 of the propyl chain, and observed H-type agonism in guinea pig ileum assays (Scheme 11).

**Scheme 12:**
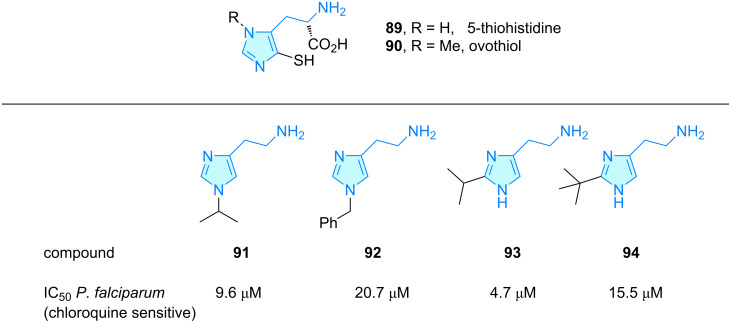
2-Aminoethylimidazole derivatives with therapeutic activity, part 4.

The sulfur-containing histidine compounds ovothiol (**90**) and thiohistidine (**89**) were evaluated for skincare anti-inflammatory properties by Brancaccio et al. (Scheme 12) [64]. These compounds, biosynthesized by microalgae, bacteria and marine invertebrates feature skin protection via Nrf2 activation (nuclear factor erythroid 2-related factor 2).

Antimalarial properties against chloroquine-sensitive and resistant *Plasmodium falciparium* strains in mice were reported by Jain and co-workers [65]. Initially, these authors developed simple halohistidine derivatives as first generation analogues showing successful in vitro antimalarial activity. Prompted by these findings, a second generation series, i.e., compounds **91**–**94** (Scheme 12), featuring simple hydrocarbon substituents was elaborated. This collection showed good activities, demonstrating the tolerance of introducing bulky moieties at position C2 or N1 of the imidazole ring. Researchers also described positive membrane diffusion features related to these changes.

The amino acid ʟ-histidine has attracted the attention of the medicinal chemistry community due to its properties not only in the aforementioned histaminergic system, but also as metal-ion chelator, proton buffering modulator, and antioxidant. Considering the importance and applications of ring-modified histidines, Sharma et al. [66] reviewed the design, synthesis, and medicinal chemistry of these motifs covering antimicrobial, antiplasmodial, CNS and anticancer applications among others.

**Scheme 13:**
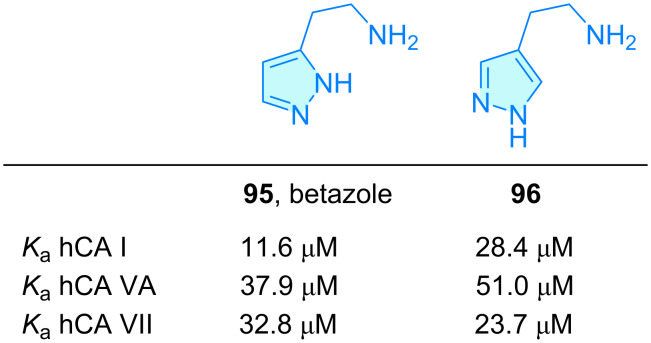
2-Aminoethylpyrazole derivatives with therapeutic activity.

**Pyrazoles:** Betazole (**95**) is a pyrazole-like histamine analogue with H_2_ receptor agonist activity (Scheme 13) and is employed as a stimulant of gastric secretion, with a 10-fold weaker activity compared to parent histamine [67]. Betazole and its isomer **96** were also found to be moderately active in the activation of human carbonic anhydrase isoforms as reported by Chiaramonte et al. (Scheme 13) [41].

**Scheme 14:**
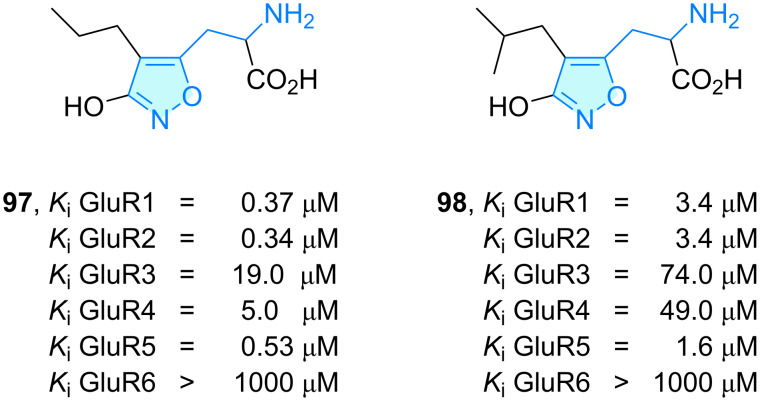
2-Aminoethylisoxazole derivatives with therapeutic activity.

**Isoxazole:** Homoibotenic acid (HIBO) analogues are known ligands with pharmacological bioactive profile towards ionotropic and metabotropic glutamate receptors (iGluR and mGluR). 4-Substituted HIBO compounds **97** and **98** (Scheme 14) portrayed by Madsen et al. [68] and Kromann et al. [69] were investigated to search new selectivity profiles. They presented different affinities towards glutamate receptors, with good potencies for the Glu1, Glu2 and Glu5 receptors. The high selectivity achievement is related to neuroprotective or neurotoxic applications following authors studies.

**Scheme 15:**
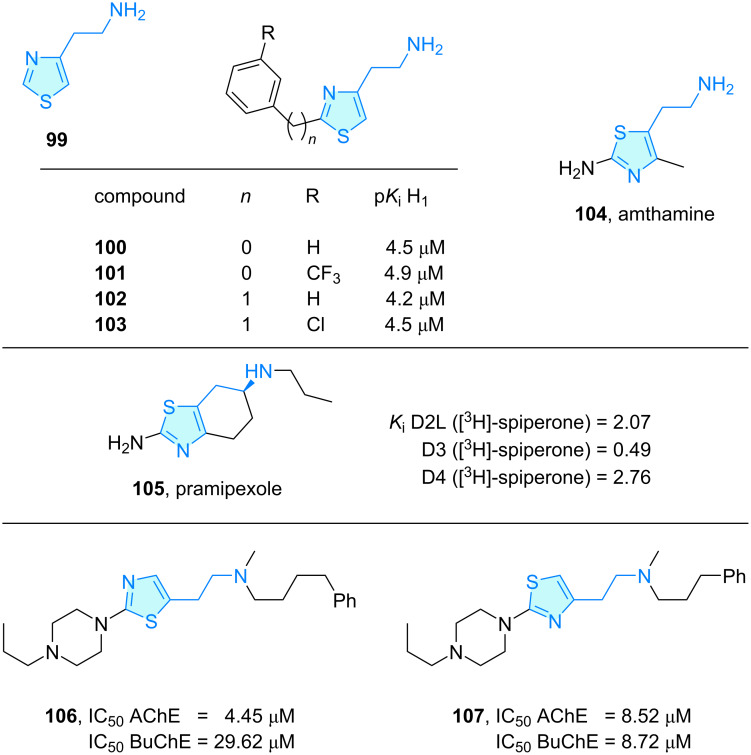
2-Aminoethylthiazole derivatives with therapeutic activity.

**Thiazoles:** 2-Thiazolylethylamine was characterized as a more selective and potent histamine H_1_ agonist [70]. Based on this, Govoni et al. [71] analyzed the pharmacological profile of several histamine H_1_ antagonists, with a section covering thiazole-based compounds. 2-(Thiazol-4-yl)ethylamine (**99**) presented a low H_1_ affinity, whereas the 2-substituted candidates **100**–**103** displayed a borderline, marginal activity (Scheme 15). A similar derivative described as a histamine H_2_ full agonist used to study gastric secretion was amthamine (**104**) [72].

Pramipexole (**105**, SND 919) is a dopamine agonist approved for the treatment of Parkinson´s symptoms like rigidity, tremor, and bradykinesia [73]. Mierau et al. [74] showed that the compound has a high affinity for the dopamine D_3_ receptor (Scheme 15).

Jonczyk and co-workers [75] evaluated a series of 1-[2-thiazol-5-yl-(2-aminoethyl)]-4-*N*-propylpiperazine and 1-[2-thiazol-4-yl-(2-aminoethyl)]-4-*N*-propylpiperazine derivatives as substrates for acetylcholinesterase (AChE) and butyrylcholinesterase (BuChE). Compounds **106** and **107** showed good inhibitory potency as multitarget-directed ligands (MTD, Scheme 15).

**Scheme 16:**
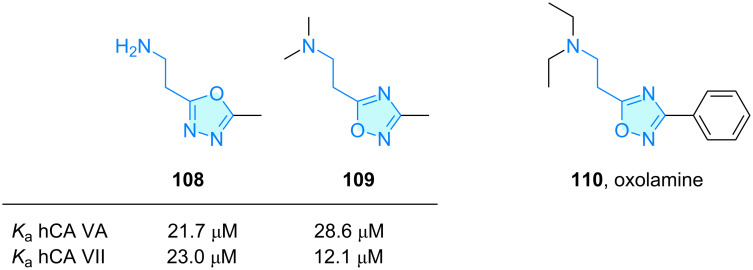
2-Aminoethyloxadiazole derivatives with therapeutic activity.

**Oxadiazole:** In their seminal work, Chiaramonte and co-workers [41] also tested 1,3,4-oxadiazole **108** and 1,2,4-oxadiazole **109** histamine congeners towards carbonic anhydrase isoforms, finding moderate potencies among them, with **109** 3 times more potent than histamine in CA type VII (Scheme 16).

Oxolamine (**110**, Scheme 16) is a cough suppressant originally synthesized by Palazzo et al. [76] and several other derivatives were developed based on ethylamine-chain homologation [77].

**Scheme 17:**
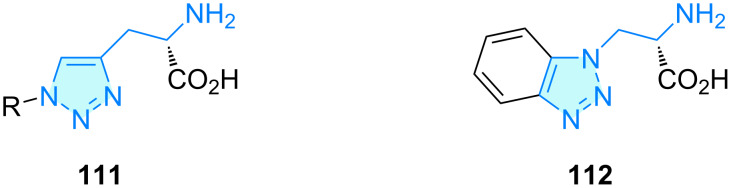
2-Aminoethyltriazole derivatives with therapeutic activity.

**Triazoles:** Hall and co-workers [78] developed 1,2,3-triazolyl analogues **111** of ʟ-histidine for ʟ-type amino acid transporter 1 (LAT1) activity, a sodium-independent membrane solute carrier protein which is used as strategic target for blood–brain-barrier drug delivery. In general, the authors found the compounds less potent than the natural substrate ʟ-tryptophan, with exception of derivative **112** (Scheme 17).

**Scheme 18:**
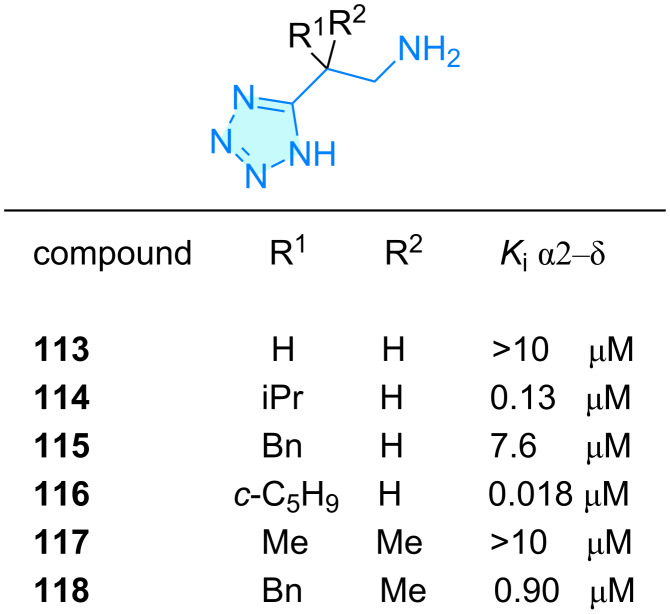
2-Aminoethyloxadiazole derivatives with therapeutic activity.

**Tetrazoles:** Taking advantage of using tetrazoles not as a phenyl-ring bioisostere, but as carboxylic acid one, Schwarz et al. [79] developed tetrazole-based pregabalin bioisosteres **113**–**118** (Scheme 18). The target protein α2-δ is involved in neurotransmitters release reduction, as a model of anxiety and neuropathic pain. In general, submicromolar affinities were observed for this family of tetrazole scaffolds.

## Conclusion

The present review focuses on an examination of the expanded 2-phenethylamine chemical space, highlighting heteroaromatic structures with reported pharmacological profiles. The close inspection of each of the phenyl and other heteroaryl ring systems reveals a conserved pattern: most of the changes are related to bioisostere structure–activity exploration of the chemical space from original phenyl hits. The results with the imidazole analogues are different, since the ʟ-histidine unit marks a non-phenyl-based scaffold hopping.

The main goal of these SAR expansions is creating new chemical matter with appealing potency and selectivity profiles. The impact of the scaffold hopping exercise in these target biomarkers depends on the nature of the targets themselves. It is noteworthy, that the use of molecular modelling tools, especially molecular docking or QSAR calculations to describe the bioisostere, impact and rationalize the observed experimental binding or efficacy measurements.

As it was stated in the introduction, only the rings described in this review, are the ones with reported activity, but there exists a plethora of other analogues containing a wide variety of heteroaryls which still need to be bioassayed. These examples, will be covered in another article on the 2-heteroaryl- (and phenyl)ethylamine series. As a final conclusion, this review of 2-heteroarylethylamines serves as an updated repository of bioisosteric rescaffolding of 2-phenethylamine derivatives evaluating affinity and aromatic core diversity.

## Data Availability

Data sharing is not applicable as no new data was generated or analyzed in this study.
